# {*N*,*N*′-Bis[(*E*)-3-phenyl­prop-2-en-1-yl­idene]propane-1,3-diamine-κ^2^ *N*,*N*′]dichloridocobalt(II)

**DOI:** 10.1107/S1600536809016274

**Published:** 2009-05-07

**Authors:** Morteza Montazerozohori, Mohammad Hossein Habibi, Mehdi Amirnasr, Keita Ariyoshi, Takayoshi Suzuki

**Affiliations:** a Department of Chemistry, Yasouj University, Yasouj 75914-353, Iran; b Catalysis Division, Department of Chemistry, University of Isfahan, Isfahan 81746-73441, Iran; c Department of Chemistry, Isfahan University of Technology, Isfahan, Iran; d Department of Chemistry, Faculty of Science, Okayama University, Tsushima-naka 3-1-1, Okayama 700-8530, Japan

## Abstract

The Co^II^ atom in the title monomeric Schiff base complex, [CoCl_2_(C_21_H_22_N_2_)], is bonded to two Cl atoms and to two N atoms of the Schiff base ligand *N*,*N*′-bis­[(*E*)-3-phenyl­prop-2-en-1-yl­idene]propane-1,3-diamine in a distorted tetra­hedral geometry. The mol­ecule has an idealised mirror symmetry, but is not located on a crystallographic mirror plane.

## Related literature

For transition metal complexes with Schiff base ligands, see: Yamada (1999 ▶). For related structures, see: Amirnasr *et al.* (2003 ▶); Blonk *et al.* (1985 ▶); Habibi *et al.* (2007*a* ▶,*b* ▶); Meghdadi *et al.* (2002 ▶); Scheidt *et al.* (1969 ▶).
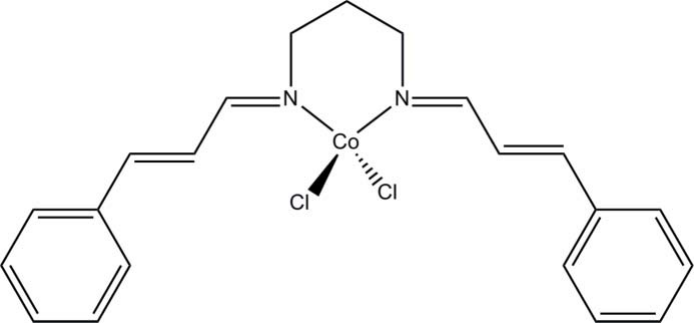


## Experimental

### 

#### Crystal data

[CoCl_2_(C_21_H_22_N_2_)]
*M* *_r_* = 432.24
Monoclinic, 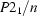

*a* = 7.4976 (5) Å
*b* = 16.1594 (8) Å
*c* = 16.6238 (10) Å
β = 91.531 (2)°
*V* = 2013.4 (2) Å^3^
*Z* = 4
Mo *K*α radiation
μ = 1.13 mm^−1^
*T* = 193 K
0.30 × 0.30 × 0.20 mm

#### Data collection

Rigaku R-AXIS RAPID diffractometer
Absorption correction: multi-scan (*ABSCOR*; Higashi, 1995 ▶) *T*_min_ = 0.729, *T*_max_ = 0.806
23596 measured reflections
5826 independent reflections
4806 reflections with *I* > 2σ(*I*)
*R*_int_ = 0.034

#### Refinement

*R*[*F* ^2^ > 2σ(*F* ^2^)] = 0.036
*wR*(*F* ^2^) = 0.091
*S* = 1.09
5826 reflections
236 parameters
H-atom parameters constrained
Δρ_max_ = 0.53 e Å^−3^
Δρ_min_ = −0.46 e Å^−3^

### 

Data collection: *PROCESS-AUTO* (Rigaku, 1998 ▶); cell refinement: *PROCESS-AUTO*; data reduction: *CrystalStructure* (Rigaku/MSC, 2004 ▶); program(s) used to solve structure: *SIR2004* (Burla *et al.*, 2005 ▶); program(s) used to refine structure: *SHELXL97* (Sheldrick, 2008 ▶); molecular graphics: *ORTEP-3* (Farrugia, 1997 ▶); software used to prepare material for publication: *SHELXL97*.

## Supplementary Material

Crystal structure: contains datablocks I, global. DOI: 10.1107/S1600536809016274/bt2939sup1.cif

Structure factors: contains datablocks I. DOI: 10.1107/S1600536809016274/bt2939Isup2.hkl

Additional supplementary materials:  crystallographic information; 3D view; checkCIF report

## Figures and Tables

**Table:** 

Co1—N1	2.0368 (13)
Co1—N2	2.0392 (13)
Co1—Cl1	2.2399 (5)
Co1—Cl2	2.2559 (5)

**Table:** 

N1—Co1—N2	93.21 (5)
N1—Co1—Cl1	117.36 (4)
N2—Co1—Cl1	118.07 (4)
N1—Co1—Cl2	106.31 (4)
N2—Co1—Cl2	106.71 (4)
Cl1—Co1—Cl2	112.965 (19)

**Fig. 1.**
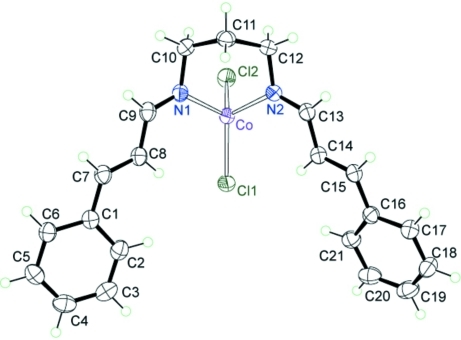
A view of the title compound, showing the atom-numbering scheme. Displacement ellipsoids are drawn at the 30% probability level and H atoms are shown as small spheres of arbitrary radii.

## supplementary crystallographic information

### Comment

Transition metal complexes with Schiff base ligands have attracted substantial interest for many years (Yamada, 1999). Cinnamaldehyde and its substituted derivatives condense with diamines to supply a range of Schiff base compounds; a small number of such bis(cinnamaldehyde)ethylenediimine ligands have been used to prepare adducts with transition metals. Among such complexes whose structures have been described are, for example, the copper(I) iodide (Habibi *et al.*, 2007*a*), (triphenylphosphine)(halogen/pseudohalogeno)- copper(I) (Habibi *et al.*, 2007*b*), copper(I) perchlorate (Meghdadi *et al.*, 2002), and the cobalt(II) chloride, cobalt(II) bromide and nickel bromide (Amirnasr *et al.*, 2003) adducts. The title complex, (I), was prepared by the reaction of CoCl_2_ with the bidentate ligand *N*,*N*'-bis[(*E*)-3-phenylprop-2-en-1-ylidene]propane-1,3-diamine (*ca*_2_pn). The molecular structure of complex (I) and the *ORTEP* structure are shown in Fig. 1. The metal centre has a tetrahedral coordination which shows signficant distortion, mainly due to the presence of the six-membered chelate ring (Table 1): the endocyclic N1—Co1—N2 angle is much narrower than the ideal tetrahedral angle of 109.5° whereas the opposite Cl1—Co1—Cl2 angle is much wider than the ideal tetrahedral angle. The Co1—Cl1 and Co1—Cl2 bond lengths are in good agreement with Co—Cl distances in other tetrahedral cobalt complexes, *e.g.* 2.229 (3) Å in Co(ethylenedimorpholine) Cl2 (Scheidt *et al.*, 1969), and 2.2434 (8) and 2.2266 (8) Å in Co[*N*,*N*-bis(3,5-dimethylpyrazol-1-ylmethyl)- aminobenzene]Cl2 (Blonk *et al.*, 1985). π-Conjugation within the azadiene fragments is consistent with the observed pattern of C—C bond distances; the predominantly double C7=C8 and C14=C15 bonds are substantially shorter than the C8—C9 and C13—C14 bonds, which have a significant π-component; the latter bonds in their turn are much shorter than the single C10—C11 and C11—C12 bonds in the propylene bridge.

### Experimental

The bidentate Schiff base ligand of *N, N'*-bis((*E*)-3-phenyl-propenylidene)-1,3-diaminopropane was synthesized by the condensation reaction of 2 mmol of (E)-3-phenypropenal and 1 mmol 1,3-diaminopropane in 10 ml dichloromethane in an ice bath for 1 h. The solution then was added drop wise to a solution of 1 mmol anhydrous CoCl_2 _in 10 ml dichloromethane under nitrogen atmosphere. The mixture was stirred for3 h and then filtered. To the filtrate, 20 ml chloroform was added and kept overnight. The crystals suitable for X-ray were filtered off and washed with chloroform (68% yield). Elemental analysis for C_21_H_22_Cl_2_CoN_2_%: Calcd.: C, 58.35; H, 5.13; N, 6.48; Found: C, 58.31; H, 5.11; N, 6.42.

### Refinement

All H atoms were placed in calculated positions and refined using a riding-model, with C—H = 0.95–0.99 Å and with *U*_iso_(H) = 1.2Ueq(C).

### Crystal data

**Table:**

[CoCl_2_(C_21_H_22_N_2_)]	*F*(000) = 892
*M**_r_* = 432.24	*D*_x_ = 1.426 Mg m^−^^3^
Monoclinic, *P*2_1_/*n*	Mo *K*α radiation, λ = 0.71075 Å
*a* = 7.4976 (5) Å	Cell parameters from 16882 reflections
*b* = 16.1594 (8) Å	θ = 3.0–29.9°
*c* = 16.6238 (10) Å	µ = 1.13 mm^−^^1^
β = 91.531 (2)°	*T* = 193 K
*V* = 2013.4 (2) Å^3^	Cubic, green
*Z* = 4	0.30 × 0.30 × 0.20 mm

### Data collection

**Table:**

Rigaku R-AXIS RAPID diffractometer	5826 independent reflections
Radiation source: fine-focus sealed tube	4806 reflections with *I* > 2σ(*I*)
graphite	*R*_int_ = 0.034
Detector resolution: 10.00 pixels mm^-1^	θ_max_ = 30.0°, θ_min_ = 3.0°
ω scans	*h* = −10→10
Absorption correction: multi-scan (*ABSCOR*; Higashi, 1995)	*k* = −22→22
*T*_min_ = 0.729, *T*_max_ = 0.806	*l* = −23→23
23596 measured reflections	

### Refinement

**Table:**

Refinement on *F*^2^	Primary atom site location: structure-invariant direct methods
Least-squares matrix: full	Secondary atom site location: difference Fourier map
*R*[*F*^2^ > 2σ(*F*^2^)] = 0.036	Hydrogen site location: inferred from neighbouring sites
*wR*(*F*^2^) = 0.091	H-atom parameters constrained
*S* = 1.09	*w* = 1/[σ^2^(*F*_o_^2^) + (0.0428*P*)^2^ + 0.5035*P*] where *P* = (*F*_o_^2^ + 2*F*_c_^2^)/3
5826 reflections	(Δ/σ)_max_ = 0.002
236 parameters	Δρ_max_ = 0.53 e Å^−^^3^
0 restraints	Δρ_min_ = −0.46 e Å^−^^3^

### Special details

**Table:**

Geometry. All e.s.d.'s (except the e.s.d. in the dihedral angle between two l.s. planes) are estimated using the full covariance matrix. The cell e.s.d.'s are taken into account individually in the estimation of e.s.d.'s in distances, angles and torsion angles; correlations between e.s.d.'s in cell parameters are only used when they are defined by crystal symmetry. An approximate (isotropic) treatment of cell e.s.d.'s is used for estimating e.s.d.'s involving l.s. planes.
Refinement. Refinement of *F*^2^ against ALL reflections. The weighted *R*-factor *wR* and goodness of fit *S* are based on *F*^2^, conventional *R*-factors *R* are based on *F*, with *F* set to zero for negative *F*^2^. The threshold expression of *F*^2^ > σ(*F*^2^) is used only for calculating *R*-factors(gt) *etc*. and is not relevant to the choice of reflections for refinement. *R*-factors based on *F*^2^ are statistically about twice as large as those based on *F*, and *R*- factors based on ALL data will be even larger.

### Fractional atomic coordinates and isotropic or equivalent isotropic displacement parameters (Å^2^)

**Table:**

	*x*	*y*	*z*	*U*_iso_*/*U*_eq_	
Co1	0.25070 (3)	0.193360 (13)	0.243448 (12)	0.02719 (7)	
Cl1	0.28692 (6)	0.05592 (2)	0.25023 (2)	0.03655 (10)	
Cl2	0.49842 (6)	0.26032 (3)	0.20514 (3)	0.03852 (11)	
N1	0.04670 (18)	0.23684 (8)	0.17195 (8)	0.0291 (3)	
N2	0.16292 (18)	0.25318 (8)	0.34289 (8)	0.0291 (3)	
C1	−0.2001 (2)	−0.01609 (10)	0.05017 (9)	0.0304 (3)	
C2	−0.1150 (2)	−0.07521 (11)	0.09975 (11)	0.0377 (4)	
H2	−0.0448	−0.0578	0.1451	0.045*	
C3	−0.1329 (3)	−0.15844 (12)	0.08302 (12)	0.0426 (4)	
H3	−0.0745	−0.1981	0.1167	0.051*	
C4	−0.2360 (3)	−0.18448 (11)	0.01710 (12)	0.0419 (4)	
H4	−0.2488	−0.2419	0.0063	0.050*	
C5	−0.3203 (2)	−0.12722 (10)	−0.03290 (11)	0.0364 (4)	
H5	−0.3900	−0.1452	−0.0781	0.044*	
C6	−0.3020 (2)	−0.04375 (10)	−0.01645 (10)	0.0330 (3)	
H6	−0.3595	−0.0045	−0.0509	0.040*	
C7	−0.1865 (2)	0.07272 (11)	0.06595 (10)	0.0325 (3)	
H7	−0.2680	0.1076	0.0373	0.039*	
C8	−0.0703 (2)	0.10972 (10)	0.11696 (9)	0.0304 (3)	
H8	0.0122	0.0768	0.1473	0.037*	
C9	−0.0678 (2)	0.19798 (10)	0.12681 (10)	0.0320 (3)	
H9	−0.1558	0.2296	0.0984	0.038*	
C10	0.0290 (2)	0.32742 (10)	0.17794 (10)	0.0342 (3)	
H10A	−0.0620	0.3469	0.1380	0.041*	
H10B	0.1440	0.3538	0.1653	0.041*	
C11	−0.0252 (2)	0.35316 (10)	0.26220 (10)	0.0340 (3)	
H11A	−0.0595	0.4123	0.2608	0.041*	
H11B	−0.1321	0.3210	0.2767	0.041*	
C12	0.1177 (2)	0.34122 (9)	0.32822 (10)	0.0342 (3)	
H12A	0.2269	0.3712	0.3131	0.041*	
H12B	0.0753	0.3659	0.3788	0.041*	
C13	0.1347 (2)	0.22441 (10)	0.41378 (9)	0.0299 (3)	
H13	0.0819	0.2604	0.4515	0.036*	
C14	0.1777 (2)	0.14149 (10)	0.43997 (9)	0.0301 (3)	
H14	0.2322	0.1041	0.4041	0.036*	
C15	0.1409 (2)	0.11698 (10)	0.51492 (10)	0.0299 (3)	
H15	0.0783	0.1550	0.5474	0.036*	
C16	0.1881 (2)	0.03716 (10)	0.55142 (9)	0.0303 (3)	
C17	0.1433 (3)	0.02236 (11)	0.63139 (10)	0.0383 (4)	
H17	0.0757	0.0621	0.6596	0.046*	
C18	0.1975 (3)	−0.05030 (12)	0.66951 (12)	0.0470 (5)	
H18	0.1663	−0.0600	0.7237	0.056*	
C19	0.2957 (3)	−0.10810 (12)	0.62942 (13)	0.0460 (5)	
H19	0.3343	−0.1572	0.6561	0.055*	
C20	0.3386 (2)	−0.09457 (11)	0.54975 (13)	0.0420 (4)	
H20	0.4057	−0.1348	0.5219	0.050*	
C21	0.2844 (2)	−0.02314 (10)	0.51072 (11)	0.0345 (3)	
H21	0.3126	−0.0149	0.4559	0.041*	

### Atomic displacement parameters (Å^2^)

**Table:**

	*U*^11^	*U*^22^	*U*^33^	*U*^12^	*U*^13^	*U*^23^
Co1	0.02769 (12)	0.02684 (12)	0.02693 (12)	0.00036 (7)	−0.00153 (8)	0.00003 (8)
Cl1	0.0445 (2)	0.02843 (19)	0.0363 (2)	0.00604 (15)	−0.00552 (17)	−0.00140 (15)
Cl2	0.0328 (2)	0.0447 (2)	0.0381 (2)	−0.00778 (17)	0.00149 (16)	0.00277 (18)
N1	0.0300 (7)	0.0313 (6)	0.0258 (6)	0.0031 (5)	−0.0003 (5)	−0.0008 (5)
N2	0.0313 (7)	0.0270 (6)	0.0287 (6)	−0.0016 (5)	−0.0015 (5)	−0.0009 (5)
C1	0.0258 (7)	0.0354 (8)	0.0300 (7)	−0.0005 (6)	0.0016 (6)	−0.0011 (6)
C2	0.0357 (9)	0.0430 (9)	0.0344 (8)	−0.0002 (7)	−0.0020 (7)	0.0037 (7)
C3	0.0413 (10)	0.0407 (10)	0.0461 (10)	0.0037 (8)	0.0050 (8)	0.0120 (8)
C4	0.0403 (10)	0.0341 (9)	0.0518 (11)	−0.0037 (7)	0.0119 (8)	−0.0011 (8)
C5	0.0299 (8)	0.0413 (9)	0.0381 (9)	−0.0053 (7)	0.0050 (7)	−0.0070 (7)
C6	0.0288 (8)	0.0394 (8)	0.0308 (8)	0.0007 (6)	0.0003 (6)	0.0001 (7)
C7	0.0285 (8)	0.0363 (8)	0.0324 (8)	0.0030 (6)	−0.0030 (6)	−0.0008 (7)
C8	0.0274 (7)	0.0356 (8)	0.0283 (7)	0.0021 (6)	0.0002 (6)	−0.0009 (6)
C9	0.0282 (8)	0.0388 (9)	0.0288 (7)	0.0036 (6)	−0.0016 (6)	−0.0010 (6)
C10	0.0386 (9)	0.0302 (8)	0.0335 (8)	0.0038 (7)	−0.0050 (7)	0.0028 (7)
C11	0.0364 (9)	0.0265 (7)	0.0390 (9)	0.0030 (6)	−0.0007 (7)	−0.0016 (7)
C12	0.0450 (10)	0.0237 (7)	0.0339 (8)	−0.0014 (6)	−0.0025 (7)	−0.0018 (6)
C13	0.0297 (8)	0.0315 (8)	0.0285 (7)	0.0010 (6)	−0.0012 (6)	−0.0030 (6)
C14	0.0307 (8)	0.0319 (7)	0.0277 (7)	0.0000 (6)	−0.0007 (6)	−0.0016 (6)
C15	0.0288 (8)	0.0325 (8)	0.0282 (7)	−0.0005 (6)	−0.0016 (6)	−0.0024 (6)
C16	0.0273 (8)	0.0332 (8)	0.0302 (7)	−0.0047 (6)	−0.0041 (6)	0.0007 (6)
C17	0.0447 (10)	0.0380 (9)	0.0322 (8)	−0.0032 (7)	−0.0010 (7)	0.0012 (7)
C18	0.0596 (13)	0.0467 (11)	0.0343 (9)	−0.0085 (9)	−0.0065 (8)	0.0105 (8)
C19	0.0425 (10)	0.0384 (9)	0.0563 (12)	−0.0044 (8)	−0.0131 (9)	0.0140 (9)
C20	0.0330 (9)	0.0337 (9)	0.0593 (12)	−0.0006 (7)	−0.0008 (8)	0.0020 (8)
C21	0.0310 (8)	0.0341 (8)	0.0384 (9)	−0.0038 (6)	0.0015 (6)	0.0022 (7)

### Geometric parameters (Å, °)

**Table:**

Co1—N1	2.0368 (13)	C10—C11	1.527 (2)
Co1—N2	2.0392 (13)	C10—H10A	0.9900
Co1—Cl1	2.2399 (5)	C10—H10B	0.9900
Co1—Cl2	2.2559 (5)	C11—C12	1.525 (2)
N1—C9	1.289 (2)	C11—H11A	0.9900
N1—C10	1.473 (2)	C11—H11B	0.9900
N2—C13	1.289 (2)	C12—H12A	0.9900
N2—C12	1.481 (2)	C12—H12B	0.9900
C1—C6	1.402 (2)	C13—C14	1.443 (2)
C1—C2	1.404 (2)	C13—H13	0.9500
C1—C7	1.462 (2)	C14—C15	1.343 (2)
C2—C3	1.379 (3)	C14—H14	0.9500
C2—H2	0.9500	C15—C16	1.465 (2)
C3—C4	1.389 (3)	C15—H15	0.9500
C3—H3	0.9500	C16—C21	1.398 (2)
C4—C5	1.385 (3)	C16—C17	1.401 (2)
C4—H4	0.9500	C17—C18	1.390 (2)
C5—C6	1.382 (2)	C17—H17	0.9500
C5—H5	0.9500	C18—C19	1.373 (3)
C6—H6	0.9500	C18—H18	0.9500
C7—C8	1.340 (2)	C19—C20	1.389 (3)
C7—H7	0.9500	C19—H19	0.9500
C8—C9	1.436 (2)	C20—C21	1.380 (2)
C8—H8	0.9500	C20—H20	0.9500
C9—H9	0.9500	C21—H21	0.9500
			
N1—Co1—N2	93.21 (5)	N1—C10—H10B	109.4
N1—Co1—Cl1	117.36 (4)	C11—C10—H10B	109.4
N2—Co1—Cl1	118.07 (4)	H10A—C10—H10B	108.0
N1—Co1—Cl2	106.31 (4)	C12—C11—C10	115.27 (14)
N2—Co1—Cl2	106.71 (4)	C12—C11—H11A	108.5
Cl1—Co1—Cl2	112.965 (19)	C10—C11—H11A	108.5
C9—N1—C10	117.61 (13)	C12—C11—H11B	108.5
C9—N1—Co1	130.55 (11)	C10—C11—H11B	108.5
C10—N1—Co1	111.78 (10)	H11A—C11—H11B	107.5
C13—N2—C12	117.02 (13)	N2—C12—C11	113.16 (13)
C13—N2—Co1	129.36 (11)	N2—C12—H12A	108.9
C12—N2—Co1	113.53 (10)	C11—C12—H12A	108.9
C6—C1—C2	118.44 (15)	N2—C12—H12B	108.9
C6—C1—C7	119.26 (14)	C11—C12—H12B	108.9
C2—C1—C7	122.30 (15)	H12A—C12—H12B	107.8
C3—C2—C1	120.34 (16)	N2—C13—C14	124.76 (15)
C3—C2—H2	119.8	N2—C13—H13	117.6
C1—C2—H2	119.8	C14—C13—H13	117.6
C2—C3—C4	120.22 (17)	C15—C14—C13	120.29 (15)
C2—C3—H3	119.9	C15—C14—H14	119.9
C4—C3—H3	119.9	C13—C14—H14	119.9
C5—C4—C3	120.42 (17)	C14—C15—C16	126.20 (15)
C5—C4—H4	119.8	C14—C15—H15	116.9
C3—C4—H4	119.8	C16—C15—H15	116.9
C6—C5—C4	119.47 (16)	C21—C16—C17	118.72 (16)
C6—C5—H5	120.3	C21—C16—C15	122.38 (15)
C4—C5—H5	120.3	C17—C16—C15	118.82 (15)
C5—C6—C1	121.10 (16)	C18—C17—C16	120.16 (18)
C5—C6—H6	119.4	C18—C17—H17	119.9
C1—C6—H6	119.4	C16—C17—H17	119.9
C8—C7—C1	126.34 (15)	C19—C18—C17	120.49 (19)
C8—C7—H7	116.8	C19—C18—H18	119.8
C1—C7—H7	116.8	C17—C18—H18	119.8
C7—C8—C9	121.45 (15)	C18—C19—C20	119.78 (17)
C7—C8—H8	119.3	C18—C19—H19	120.1
C9—C8—H8	119.3	C20—C19—H19	120.1
N1—C9—C8	123.85 (15)	C21—C20—C19	120.49 (18)
N1—C9—H9	118.1	C21—C20—H20	119.8
C8—C9—H9	118.1	C19—C20—H20	119.8
N1—C10—C11	111.07 (13)	C20—C21—C16	120.33 (17)
N1—C10—H10A	109.4	C20—C21—H21	119.8
C11—C10—H10A	109.4	C16—C21—H21	119.8
			
N2—Co1—N1—C9	−125.65 (15)	C10—N1—C9—C8	−178.17 (15)
Cl1—Co1—N1—C9	−1.63 (16)	Co1—N1—C9—C8	−1.2 (3)
Cl2—Co1—N1—C9	125.90 (14)	C7—C8—C9—N1	−176.41 (17)
N2—Co1—N1—C10	51.43 (11)	C9—N1—C10—C11	112.10 (16)
Cl1—Co1—N1—C10	175.45 (9)	Co1—N1—C10—C11	−65.40 (15)
Cl2—Co1—N1—C10	−57.02 (11)	N1—C10—C11—C12	69.65 (18)
N1—Co1—N2—C13	128.77 (14)	C13—N2—C12—C11	−118.87 (16)
Cl1—Co1—N2—C13	5.30 (16)	Co1—N2—C12—C11	58.15 (16)
Cl2—Co1—N2—C13	−123.14 (14)	C10—C11—C12—N2	−65.56 (19)
N1—Co1—N2—C12	−47.80 (11)	C12—N2—C13—C14	−177.22 (14)
Cl1—Co1—N2—C12	−171.27 (9)	Co1—N2—C13—C14	6.3 (2)
Cl2—Co1—N2—C12	60.29 (11)	N2—C13—C14—C15	−179.13 (16)
C6—C1—C2—C3	−0.3 (3)	C13—C14—C15—C16	−175.37 (14)
C7—C1—C2—C3	179.37 (17)	C14—C15—C16—C21	1.8 (3)
C1—C2—C3—C4	−0.3 (3)	C14—C15—C16—C17	178.41 (16)
C2—C3—C4—C5	0.7 (3)	C21—C16—C17—C18	1.4 (3)
C3—C4—C5—C6	−0.4 (3)	C15—C16—C17—C18	−175.39 (16)
C4—C5—C6—C1	−0.2 (3)	C16—C17—C18—C19	0.2 (3)
C2—C1—C6—C5	0.6 (2)	C17—C18—C19—C20	−1.2 (3)
C7—C1—C6—C5	−179.12 (15)	C18—C19—C20—C21	0.5 (3)
C6—C1—C7—C8	−166.72 (17)	C19—C20—C21—C16	1.1 (3)
C2—C1—C7—C8	13.6 (3)	C17—C16—C21—C20	−2.0 (2)
C1—C7—C8—C9	179.17 (16)	C15—C16—C21—C20	174.62 (16)
